# Blood Concentration of Macro- and Microelements in Women Who Are Overweight/Obesity and Their Associations with Serum Biochemistry

**DOI:** 10.3390/life14040465

**Published:** 2024-04-02

**Authors:** Zuzana Knazicka, Maros Bihari, Ivona Janco, Lubos Harangozo, Julius Arvay, Anton Kovacik, Peter Massanyi, Branislav Galik, Jorge M. A. Saraiva, Marta Habanova

**Affiliations:** 1 Institute of Nutrition and Genomics, Faculty of Agrobiology and Food Resources, Slovak University of Agriculture, Trieda Andreja Hlinku 2, 94976 Nitra, Slovakia; zuzana.knazicka@uniag.sk (Z.K.); xbihari@uniag.sk (M.B.); branislav.galik@uniag.sk (B.G.); 2 AgroBioTech Research Center, Slovak University of Agriculture, Trieda Andreja Hlinku 2, 94976 Nitra, Slovakia; ivona.janco@uniag.sk; 3 Institute of Food Sciences, Faculty of Biotechnology and Food Sciences, Slovak University of Agriculture, Trieda Andreja Hlinku 2, 94976 Nitra, Slovakia; lubos.harangozo@uniag.sk (L.H.); julius.arvay@uniag.sk (J.A.); 4 Institute of Applied Biology, Faculty of Biotechnology and Food Sciences, Slovak University of Agriculture, Trieda Andreja Hlinku 2, 94976 Nitra, Slovakia; anton.kovacik@uniag.sk (A.K.); peter.massanyi@uniag.sk (P.M.); 5 LAQV-REQUIMTE, Department of Chemistry, Campus Universitário de Santiago, University of Aveiro, 3810-193 Aveiro, Portugal; jorgesaraiva@ua.pt

**Keywords:** risk elements, blood serum, biomarkers, women, overweight, obesity, anthropometric parameters, health

## Abstract

Risk elements in blood matrices can affect human health status through associations with biomarkers at multiple levels. The aim of this study was to analyze 15 macro- and microelements in the blood serum of women with overweight (BMI of ≥25 kg/m^2^) and obesity (BMI of ≥30 kg/m^2^) and to examine possible associations with biochemical, liver enzymatic parameters, and markers of oxidative stress. Based on the power calculation, the study involved women (in the postmenopausal stage) with overweight (*n* = 26) and obesity (n = 22), aged between 50–65 years. Multifrequency bioelectrical impedance analysis was used to measure body composition parameters. Concentrations of elements were determined by inductively coupled plasma optical emission spectrometry, and Hg was measured using cold-vapor atomic absorption spectroscopy. Individuals with obesity, as indicated by a higher BMI, percentage of body fat, and visceral fat area, had elevated serum levels of Ca, Mg, Fe, Al, Sr, Pb, and Hg. Concentrations of Al, Cu, K, Sb, Zn, and Pb significantly affected biochemical and liver function markers in women with overweight or obesity. Elements such as Cu and Al were associated with increased total cholesterol. The correlation analysis between total antioxidant status and Cu, Al, and Ni confirmed associations in both groups. Our findings underscore the importance of addressing excess body weight and obesity in relation to risk elements. The results of the research could be beneficial in identifying potential targets for the treatment or prevention of comorbidities in people with obesity.

## 1. Introduction

Investigation of heavy or trace elements has remained one of the primary focuses of toxicological studies in recent years [1,2,3,4]. Elements can interact with various biomarkers at multiple levels [5,6]. The human body utilizes inorganic compounds, including mineral elements, to support a variety of biological and physiological processes [7,8]. Elements can be classified into essential and non-essential (or toxic) based on their health effects [9]. Essential elements crucial for human nutrition include potassium (K), sodium (Na), chloride (Cl), calcium (Ca), selenium (Se), manganese (Mn), iodine (J), chromium (Cr), cobalt (Co), molybdenum (Mo), fluorine (F), nickel (Ni), silicon (Si), iron (Fe), copper (Cu), zinc (Zn), and boron (B). These elements serve as crucial cofactors in the structure of specific enzymes and are indispensable in various biochemical processes [4,8]. They are divided into two categories: macroelements [K, Na, Ca, magnesium (Mg), and phosphorus (P)], which are present in concentrations exceeding 0.01% of total body mass and mediate structural and regulatory functions such as fluid balance, bone and tooth formation, nerve transmission, and oxygen transport [8,10]. The second group, microelements (or trace elements) in adults, are present in much lower concentrations yet play crucial roles in numerous metabolic processes and in maintaining healthy immune functions [10]. Non-essential elements such as lead (Pb), arsenic (As), cadmium (Cd), and mercury (Hg) exhibit toxic effects even at very low concentrations, posing a significant risk to human health [9,11,12]. Although each toxic element exhibits unique toxicological properties, common symptoms include oxidative damage, disruption in cellular and enzymatic mechanisms, and the formation of adducts with deoxyribonucleic acid or proteins [13]. Deviations from optimal levels of elements can correlate with a variety of diseases. Thus, understanding the associations of elements in biological matrices may enhance the diagnosis and treatment of diseases. Developing reliable analytical methods to determine macro- and microelement levels in samples is crucial for creating biological models that deepen our understanding of the relationship between elements and diseases [14].

Obesity and associated metabolic disorders are global public health concerns [15,16,17]. Currently, the prevalence of obesity is increasing across all age groups and in both sexes, irrespective of geographical location, ethnicity, or socioeconomic status [18]. According to current trends, it is predicted to affect more than one billion adults, or one-sixth of the world’s population, by 2030 [19]. The World Obesity Federation’s Global Obesity Observatory [20] has provided a comprehensive overview of rates of overweight and obesity in the Slovak population from 2000 to 2019. The prevalence of overweight among Slovak adults (aged 18 years and older) reached approximately 39% in 2019, with obesity affecting more than 19% of this population. Among these, the rates of obesity were 20.80% for men and 18.70% for women.

The World Health Organization (WHO) defines obesity and overweight as abnormal or excessive body fat accumulation that may have negative health effects [21]. Discussing obesity, it is essential to introduce the concept of Body Mass Index (BMI), with a BMI of ≥25 kg/m^2^ generally considered overweight and a BMI of ≥30 kg/m^2^ classified as obesity [22,23]. This index is the most commonly used criterion for identifying individuals with overweight or obesity [24], and it is applicable to both sexes and all adult age groups. However, it should be considered basic guidance, as it does not capture the proportion of fat mass and fat-free mass or the changes in these compartments among individuals [25,26,27]. Similarly, Wu et al. [28] and Bihari et al. [24] reported that evaluating adults with overweight or obesity requires not only BMI but also other diagnostic elements. The percentage of body fat (PBF; %) may offer a more reliable and accurate indicator compared to BMI when assessing overweight or obesity [29,30,31]. Furthermore, attention should also be focused on the visceral fat area (VFA; cm^2^), which appears to be a valuable parameter for diagnosing multifactorial diseases and assessing associated health risks [24,27,32]. For most of the population, obesity results from a complex interplay of an individual’s genetic predisposition and various biological, behavioral, psychosocial, socioeconomic, and environmental factors (including chronic stress) [33]. High-energy-dense diets, sedentary lifestyles, low physical activity, and eating disorders are key risk factors [34]. Furthermore, macro- and micronutrients have been identified as crucial in regulating metabolic processes, contributing to the etiology of this disease [35]. Obesity is associated with a wide range of comorbidities, such as cardiovascular disease, hypertension, stroke, various chronic diseases, including type 2 diabetes mellitus, gallbladder disorders, dyslipidemia, osteoarthritis, gout, and various pulmonary diseases, notably sleep apnea, among many other health complications. Additionally, a link has been identified between obesity and several types of cancer [23,36,37,38,39].

Blood analysis serves as a valuable alternative for evaluating health status [5]. Parameters, such as serum biochemistry and enzymatic markers, may offer insights into the physiological responses of the organism in relation to various elements [6]. To our knowledge, there are currently a limited number of human blood studies involving participants with overweight or obesity and that have utilized inductively coupled plasma optical emission spectrometry (ICP-OES) for the analysis of risk elements. Our primary goal was to determine the concentrations of 15 macro- and microelements in the blood serum of women with overweight/obesity (in the postmenopausal phase) and to investigate potential associations between selected markers and risk elements. Specifically, we focused on the quantitative composition of these essential, potentially toxic, and toxic elements (mg/mL): aluminum (Al), barium (Ba), Ca, Cu, Fe, K, Mg, Na, Ni, Pb, antimony (Sb), Se, strontium (Sr), and Zn, using ICP-OES. Furthermore, we determined the total Hg concentration (ng/µL) using cold-vapor atomic absorption spectroscopy (CV-AAS). Our subsequent objective was to assess anthropometric parameters (body weight, body height, BMI, PBF, VFA, and systolic and diastolic blood pressures), biochemical and enzymatic parameters [serum total cholesterol (TC), glucose, aspartate aminotransferase (AST), alanine aminotransferase (ALT), γ-glutamyltransferase (GGT), total bilirubin (tbil), direct/conjugated bilirubin (dbil)], and total antioxidant status (TAS).

## 2. Materials and Methods

### 2.1. Characteristics of Participants

Before recruiting subjects, we conducted a power analysis to determine the necessary number of volunteers, ensuring that the obtained results would be meaningful. We aimed for a total sample size with sufficient power (80%). The present study included 51 volunteers, adult women from the staff of the Slovak University of Agriculture (SUA) in Nitra (Slovakia), who underwent eligibility screening for participation in the study. Forty-eight (*n* = 48) non-medicated women with overweight (*n* = 26) and obesity (*n* = 22), with a mean age of 56.92 years, were enrolled in the study. The volunteers had to meet the following inclusion criteria: an age range of 50–65 years (in the postmenopausal stage), a BMI of ≥25 kg/m^2^ (women with overweight), and a BMI of ≥30 kg/m^2^ (women with obesity) as defined by the WHO [21,22]. Additionally, participants were required to maintain a constant body weight (±3 kg) over the last 3 months and limit alcohol consumption to ≤30 g/day. Individuals were excluded if they had a history of diabetes mellitus, cardiovascular and cerebrovascular diseases, uncontrolled hypertension treated with medication, were using medication for weight loss, cholesterol-lowering medications or supplements, had gastrointestinal tract disorders, undergone gastrointestinal surgery, had chronic hepatitis, renal diseases, cancer, thyroid abnormalities, were regular smokers, or had alcohol or drug addiction. Of the total registered volunteers (*n* = 51), three participants did not meet these criteria and were subsequently excluded from the study.

This study was carried out according to the guidelines of the Declaration of Helsinki and approved by the Ethical Committee of the Specialized Hospital of St. Svorad Zobor in Nitra, Slovakia (protocol No. 4/071220/2020), as well as by the SUA in Nitra, Institute of Nutrition and Genomics, Slovakia. All participants provided written informed consent prior to their participation in the study.

### 2.2. Anthropometric Measurements

Trained personnel carried out anthropometric measurements. Body weight (kg) and height (cm) were measured using outpatient electronic medical scales (Tanita WB-3000, Tanita Co., Tokyo, Japan). BMI was calculated by dividing the body weight in kilograms (kg) by the square of the height in meters (m^2^). PBF (%) and VFA (cm^2^) were determined using multifrequency bioelectrical impedance analysis (MF-BIA) with the InBody 720 analyzer (Biospace Co., Ltd., Seoul, Republic of Korea). Participants were provided with information regarding the procedure and informed about the risks of MF-BIA measurement in the case of an electrical device implanted in the body on the heart. The measurements were conducted in a controlled laboratory setting, adhering to the manufacturer’s guidelines. Systolic and diastolic blood pressures (mm Hg) were measured in duplicate using the digital upper arm electronic monitor (Omron M7 Intelli IT, HEM-7361T-EBK, Omron Healthcare Co., Ltd., Tokyo, Japan). Participants were instructed to maintain a seated and calm posture, ensuring they had rested for a minimum of 15 min prior to each measurement [29,40]. All anthropometric characteristics were recorded as the average values.

### 2.3. Biological Material

The blood (*n* = 48) for biochemical analysis was collected in the morning after 8 h of fasting from the participants. Venous blood from the peripheral vein was collected in a standard manner by a qualified person using 2.50 mL tubes containing ethylenediaminetetraacetic acid (EDTA) and 7.50 mL serum gel tubes. Once the blood fractions were separated and centrifuged in serum gel tubes at 1800 rpm for 15 min at 10 °C (Hettich^®^ MIKRO 220R, Andreas Hettich GmbH & Co., Tuttlingen, Germany), the blood serum samples were stored at −80 °C until analysis [40].

### 2.4. Analysis of Biochemical Parameters

The selected biochemical markers, such as serum TC (mmol/L), glucose (mmol/L), and enzymatic parameters including AST (µkat/L), ALT (µkat/L), GGT (µkat/L), tbil (µmol/L), and dbil (µmol/L), were determined in the biochemical laboratory of the Specialized Hospital of St. Svorad Zobor in Nitra (Slovakia). These parameters were analyzed using standard commercial DiaSys kits (Diagnostic Systems GmbH, Holzheim, Germany) on the ultra-compact automated clinical-chemistry analyzer BioMajesty^®^ JCA-BM6010/C (JEOL Ltd., Tokyo, Japan). TAS (mmol/L) was determined from thawed samples and analyzed in the biochemical laboratory of the Institute of Nutrition and Genomics (SUA in Nitra, Slovakia), using a Biolis 24i Premium (Tokyo Boeki Machinery Ltd., Tokyo, Japan) with commercially available diagnostic Randox reagents (Randox Laboratories Ltd., Crumlin, UK).

### 2.5. Analysis of Elements in Blood Serum

The concentrations of macro- and microelements (Al, Ba, Ca, Cu, Fe, K, Mg, Na, Ni, Pb, Sb, Se, Sr, and Zn) in the blood serum were determined by the ICP-OES. 

Initially, a pre-analytical procedure was conducted. All chemicals utilized in sample preparation were of high purity and intended for trace-select analysis. The samples (1.0 mL) were mineralized in a high-performance microwave digestion system ETHOS-One (Milestone Srl., Sorisole, BG, Italy), in a solution of 5.0 mL nitric acid (HNO_3_; ≥69%; for trace analysis; from Lambda Life s r.o. Bratislava, Slovakia; producer: Sigma-Aldrich Chemie GmbH, Steiheim, Germany) and 1.0 mL hydrogen peroxide (H_2_O_2_; ≥30%; for trace analysis; from Lambda Life s r.o. Bratislava, Slovakia; producer: Sigma-Aldrich Chemie GmbH, Steiheim, Germany), which were added directly to the PTFE vessels. The obtained samples, including the blank sample, underwent digestion following the manufacturer’s recommended tissue method to ensure the attainment of the most dependable results. The method included heating and cooling phases (the heating phase: heating to 200 °C over 15 min and maintaining at 200 °C for 15 min; the cooling phase: active cooling to achieve a temperature of 50 °C in 15 min. Through a quantitative Whatman^®^ filter paper Grade No. 595 (basis weight 68 g/m^2^, thickness 150 μm, pore size 4–7 μm; VWR International, Leuven, France), the digested samples were filtered into 50 mL volumetric flasks and filled with deionized water (ddH_2_O; 18.2 MΩ cm^−1^; 25 °C, Synergy UV, Merck Millipore, Guyancourt, France) to the final volume. Sample solutions were stored in polyethylene tubes until ICP-OES analysis [4,41]. 

Micro- and macroelements in the blood serum were quantified using an inductively coupled plasma-optical emission spectrometer Agilent ICP-OES 720 (Agilent Technologies Inc., Santa Clara, CA, USA), with an axial plasma configuration equipped, and an auto-sampler, the SPS-3 (Agilent Technologies, GmbH, Basel, Switzerland). The detailed experimental conditions are described in Table 1. Limits of detection (LoD; μg/L) for measured elements in serum (mg/mL) were as follows: Ag 0.30; Al 0.20; As 1.50; Ba 0.03; Ca 0.01; Cd 0.05; Co 0.20; Cr 0.15; Cu 0.30; Fe 0.10; K 0.30; Li 0.06; Mg 0.01; Mn 0.03; Na 0.15; Ni 0.30; Pb 0.80; Sb 2.00; Se 2.00; Sr 0.01; and Zn 0.20. The limit of quantification (LoQ; μg/L) for measured elements in serum (mg/mL) were as follows: Ag 0.99; Al 0.66; As 4.95; Ba 0.10; Ca 0.03; Cd 0.17; Co 0.66; Cr 0.50; Cu 0.99; Fe 0.33; K 0.99; Li 0.20; Mg 0.03; Mn 0.10; Na 0.50; Ni 0.99; Pb 2.64; Sb 6.60; Se 6.60; Sr 0.03; and Zn 0.66. All samples were analyzed in triplicate for the concentration of 21 elements. In the study, Multielement Standard Solution V for ICP (Sigma-Aldrich Production, GmbH, Buchs, Switzerland) was used. Argon and carbon were used as internal standard elements. The validity of the entire method was confirmed through the use of certified reference material (CRM) ERM^®^-CE278k (muscle tissue; IRMM, Geel, Belgium). The results of the analysis were expressed in mg/mL.

The total Hg concentration in blood serum samples was determined using CV-AAS on the AMA 254 Hg analyzer (Altec spol. s r.o., Prague, Czech Republic), with a detection limit of 1.50 ng/kg DM (dry matter). The analysis was performed directly on the sample (100 µL) without pretreatment. Quantitative Hg determination occurred at λ = 253.65 nm. CRM materials ASTASOL^®^ (Czech Metrology Institute, Brno, Czech Republic; CZ 9024 (1N) Hg) were assessed to verify the quality of the measurements. The CRM measurement was performed six times [42]. The obtained results were expressed as ng/µL.

### 2.6. Statistical Analysis

Power calculations were used to determine the appropriate number of participants for the study using G*Power 3.1.9.7 software [43]. The minimum power (1-β error probability, with α = 0.05) required for our study was set at 80%. Before conducting statistical analysis, the collected data underwent normality testing using the Kolmogorov-Smirnov test. Results are presented as the arithmetic mean ± standard deviation (SD). Other descriptive characteristics are also presented (minimum, maximum, and coefficient of variation). Thereafter, significant differences between selected groups were evaluated using an unpaired *t*-test for parametric data. The Spearman R correlations were used to determine mutual associations between element levels and all parameters tested in the blood serum of participants. We also evaluated the relationships between macro- and microelements as well as biomarkers in groups of women with overweight or obesity. Statistical software GraphPad Prism 6.01 (GraphPad Software Incorporated, San Diego, CA, USA) and STATGRAPHICS Centurion (© StatPoint Technologies, Inc., Warrenton, VA, USA) were used for statistical evaluation. Statistical significance was set at three levels: *** (*p* < 0.001); ** (*p* < 0.01); * (*p* < 0.05).

## 3. Results

The study included 48 women, ranging in age from 50 to 65 years, with a mean age of 56.92 years. The descriptive characteristics of all participants are presented in Table 2. Regarding body composition parameters, the average values of PBF and VFA calculated for all participants did not fall below the reference range. In the assessment of PBF, it was observed that women with obesity had higher values compared to those classified as overweight (44.86 ± 4.31% and 38.74 ± 2.83%, respectively, *p* < 0.001). The average values of VFA were 120.03 ± 13.08 cm^2^ for women who were overweight and 148.90 ± 18.97 cm^2^ for women who with obesity. Blood pressure measurements exceeded normal levels (≤120/80 mmHg) in both monitored groups; however, they remained below the hypertension cutoff of 140/90 mmHg.

The average concentrations of biochemical and liver enzymatic parameters, as well as TAS of blood serum in all monitored groups, are reported in Table 3. Regarding metabolic characteristics, all participants exhibited high or higher levels of serum TC (6.17 ± 1.04 mmol/L; *p* > 0.05 for women with overweight and 6.58 ± 0.91 mmol/L; *p* > 0.05 for women with obesity) compared to the reference value (<5.20 mmol/L) [45]. Concentrations of glucose, tbil, and dbil in both groups exceeded normal levels. The liver function biomarkers for all participants were within the reference range; however, levels of ALT and GGT were significantly higher in women with obesity. Identical values of TAS were detected in both monitored groups, falling within the optimal reference range (1.30–1.77 mmol/L) [46].

In the present study, blood serum was used as a matrix to quantify macro- and microelements. The minimum and maximum concentrations of risk elements are presented in Table 4. For women with overweight, the general trend of decreasing element levels in the serum was as follows: Na > K > Ca > Mg > Fe > Al > Cu > Zn > Sr > Ba > Se > Sb > Ni > Pb > Hg. Sodium was the most frequently detected macroelement in both groups. The lowest concentration was determined for Hg, measured by the CV-AAS method. In the blood serum samples of women with obesity, the scheme was as follows: Na > Ca > K > Mg > Fe > Al > Cu > Zn > Sr > Ba > Se > Sb > Pb > Ni > Hg. Differences were noted for Ca, Mg, Fe, Al, Sr, Pb, and Hg, with higher levels of these elements observed in women with obesity. Concentrations of Ag, As, Cd, Co, Cr, Li, and Mn were below the detection limits of the method.

Statistically significant positive associations were observed between glucose and liver enzymes, AST (r = 0.4529; *p* < 0.05) and ALT (r = 0.5334; *p* < 0.01), as presented in Table 5. Moderate positive correlations were found between TC and tbil (r = 0.4575; *p* < 0.05), as well as between AST and ALT (r = 0.5693; *p* < 0.01) in women with overweight. A strong, statistically significant correlation was detected between tbil and dbil (r = 0.9139; *p* < 0.001). For women with obesity, positive correlations were confirmed between tbil and dbil (r = 0.8916; *p* < 0.001) and between AST and ALT (r = 0.7652; *p* < 0.001) (Table 6).

Associations between macro- and microelements in the blood serum of women who are overweight are presented in Table 7. The analysis revealed strong positive correlations between Ca and Mg (r = 0.9285; *p* < 0.001), Ca and Sr (r = 0.9515; *p* < 0.001), Ca and Al (r = 0.7623; *p* < 0.01), Mg and Sr (r = 0.8777; *p* < 0.001), and Al and Sr (r = 0.6789; *p* < 0.01). Moderate, statistically significant (*p* < 0.01) positive correlations were confirmed between Ca and Ba (r = 0.6254), Mg and Al (r = 0.6103), Mg and Ba (r = 0.5785), and Ba and Sr (r = 0.6623). Sodium is positively correlated with Mg (r = 0.4523; *p* < 0.05) and Hg (r = 0.5036; *p* < 0.05). The analysis also revealed significant negative associations between Se and Al (r = −0.6152; *p* < 0.01) and Ba and Ni (r = −0.5833; *p* < 0.05). For women with obesity, statistically significant (*p* < 0.001) strong positive correlations were found between Na and Ca (r = 0.7764), Mg and Ca (r = 0.8938), Sr and Ca (r = 0.9187), Na and Mg (r = 0.7154), Na and Sr (r = 0.7290), as well as Mg and Sr (r = 0.9176). Significant, positive correlations were also observed between Ba and Ca, Zn and Ca, Na and K, Fe and K, Mg and Ba, Mg and Zn, Mg and Sb, Ba and Sr, as well as Cu and Ni. Selenium showed strong or moderate, statistically significant negative correlations with various risk elements (Table 8). 

Statistically significant correlations between risk elements and all investigated biomarkers in monitored groups are presented in Table 9. The correlation analysis in the blood serum of women who were overweight showed significant positive associations between Al and TC (r = 0.5441), Pb and GGT (r = 0.6263), and Cu and TAS (r = 0.4055). The levels of Zn were negatively correlated with tbil (r = −0.5400) and dbil (r = −0.5243). For women with obesity, a statistically significant positive correlation was found between Cu and TC (r = 0.5530). Furthermore, the correlation analysis confirmed significant positive associations between TAS and Al (r = 0.5939), as well as Ni (r = 0.6485). Liver enzymes, such as AST, positively correlated with K (r = 0.4529) and ALT with Sb (r = 0.5241).

## 4. Discussion

Our previous studies [24,29,51] confirmed that body composition analysis should be the basis for assessing obesity risk. In the present study, we found that women with obesity had a higher body composition parameter PBF compared to women with overweight (44.86 ± 4.31% and 38.74 ± 2.83%, respectively, *p* < 0.001). The average values of VFA were 120.03 ± 13.08 cm^2^ for women with overweight and 148.90 ± 18.97 cm^2^ for women with obesity, exceeding the reference level (<100 cm^2^). Jeon et al. [52] found that VFA positively correlated with BMI, blood pressure, and biochemical parameters, as well as other components of metabolic syndrome. Furthermore, they suggest that combining BMI assessment with VFA determination by the BIA method could serve as a method for predicting the risk of metabolic syndrome. Zając-Gawlak et al. [53] demonstrated that postmenopausal women with VFA > 100 cm^2^ have a 12 times higher risk of developing metabolic syndrome compared to those women with VFA < 100 cm^2^. 

Clinical research suggests that excess body weight and obesity are linked to metabolism malfunctions and are associated with alterations in the levels of mineral elements in the body [54,55]. The present study revealed associations between serum concentrations of macro- and microelements and biochemistry markers in women with overweight/obesity. The general trend of decreasing serum concentrations of elements in the group of women with obesity was as follows: Na > Ca > K > Mg > Fe > Al > Cu > Zn > Sr > Ba > Se > Sb > Pb > Ni > Hg. Differences were noted for Ca, Mg, Fe, Al, Sr, Pb, and Hg, with higher levels of these elements in women with obesity. Sodium emerged as the predominant macroelement observed in both groups. It has been linked to an increased risk of obesity [56], which may affect the metabolism of insulin and glucose, accelerate leptin production or secretion, and enhance leptin resistance. This can lead to an energy imbalance and the accumulation of adipose tissue mass [57]. Our results demonstrate statistically significant (*p* < 0.001) strong positive correlations between Na and Mg (r = 0.7154), as well as between Na and Sr (r = 0.7290), in women with obesity. An important metabolic disturbance in individuals with obesity is adipose tissue inflammation, and Mg deficiency appears to activate proinflammatory pathways [58]. The association between individuals with excess body weight and Sr levels has not been thoroughly investigated; therefore, it requires further research. Furthermore, our analysis revealed a significant (*p* < 0.001) association between serum Ca levels and Na (r = 0.7764), Mg (r = 0.8938), and Sr (r = 0.9187) in women with obesity. Strong, statistically significant (*p* < 0.001), positive correlations were confirmed between Ca and Mg (r = 0.9285), Ca and Al (r = 0.7623), and Ca and Sr (r = 0.9515) in women with overweight. Recently, clinical observations have confirmed a significant association between metabolic syndrome and increased serum Ca levels in adults with overweight and obesity [59]. Previous studies have reported inconsistent results regarding the association between obesity and serum Ca levels, with some indicating a positive correlation, while others report an inverse correlation [60,61].

In our study, the concentrations of Ag, As, Cd, Co, Cr, Li, and Mn in the human blood matrix were determined to be below the detection limits for the ICP-OES method. Harrington et al. [14] confirmed that some elements can be problematic for human analysis with this method. As a result, elements such as Cr, Co, Mo, and Mn were identified at relatively low levels.

It is important to take into account that the results obtained using the ICP-OES method provide concentrations of total elements found in the samples without accounting for the species of the element present. This distinction is particularly significant for Zn, Fe, and Cu. The human body has evolved various biochemical mechanisms to sequester elements and minimize the potential toxicological impact posed by free ions. These mechanisms often involve binding excess elements with proteins or small molecules to prevent chemical reactions. Some examples of this principle include transferrin and metallothioneins [62,63]. According to Lecube et al. [64], postmenopausal women with obesity had higher levels of the soluble transferrin receptor than non-obese postmenopausal women. Menzie et al. [65] observed significantly lower levels of serum Fe and transferrin saturation in adults with obesity compared to individuals without obesity. Additionally, fat mass was identified as a significant negative predictor of serum Fe concentration. Our correlation analysis showed a positive association between Fe and Ni (r = 0.5049; *p* < 0.05) for women with overweight and between Fe and K (r = 0.4907; *p* < 0.05) for women with obesity. Some studies indicate a link between obesity and Fe deficiency anemia, potentially resulting from elevated hepcidin levels caused by chronic inflammation [66]. 

Copper is important for antioxidation processes, serves as a coenzyme in mitochondrial homeostasis, and is involved in inflammatory responses as well as Fe metabolism [67]. Obesity is associated with an imbalance in Cu levels [55]. Disturbances in Cu metabolism may trigger hypercholesterolemia by increasing the production of reactive oxygen species (ROS), causing oxidative stress, and leading to the oxidation of low-density lipoproteins [68]. This effect of increased serum Cu intensifies the unfavorable impact of excess body weight on health and appears to be one of the many mechanisms linking obesity with oxidative stress and atherosclerosis [55]. In our study, we found a moderate, statistically significant positive correlation between Cu and increased serum TC levels (r = 0.5530) in women with obesity, and an association with TAS (r = 0.4055) in women with overweight. Our results proved a moderate positive correlation between Al and TC (r = 0.5441) in the blood serum of women with overweight, which could disrupt metabolic processes. Aluminum is known to displace Fe from important enzymatically active proteins, resulting in dysfunctional mitochondria geared towards lipogenesis rather than energy production. Additionally, Al toxicity leads to an increase in very low-density lipoprotein secretion and a decrease in β-oxidation of fatty acids [69]. This fact is directly linked to the accumulation of fatty tissue seen in obesity [70].

Generally, elements generate and promote the overproduction of ROS [71]. We observed many significant correlations between TAS and elements, such as Al (r = 0.5939), and Ni (r = 0.6485), in women with obesity. Shi et al. [72] confirmed that Ni toxicity is associated with ROS generation, subsequent lipid peroxidation, and alkyl and alkoxyl radical production. Additionally, oxidative stress can disrupt the balance of glutathione reductase and the mitochondrial antioxidant defense system through the formation of Ni-mercaptan complexes [73,74]. 

The total bilirubin value is a sensitive indicator of liver damage. Kipp et al. [75] found that bilirubin negatively correlated with BMI and adiposity in individuals with obesity compared to those without obesity. Our results proved a moderate negative correlation between Zn and tbil (r = −0.5400), and dbil (r = −0.5243) in women with overweight. Zinc plays an important role in regulating zinc-α2-glycoprotein (ZAG) homeostasis, which is essential for lipid metabolism and homeostasis of glucose. The primary biological function of ZAG involves the mobilization of lipids, particularly in white adipose tissues [76]. Additionally, the authors demonstrated that excess body weight is negatively associated with Zn levels in both erythrocytes and plasma. The clinical study of Payahoo et al. [77] indicated that Zn supplementation could lead to improvements in BMI, body weight, and triglyceride, without considerable effects on the lipid profile and glucose levels.

The blood levels of elements have been significantly associated with liver function parameters [78,79]. Our present study observed an association between certain elements and liver function biomarkers. We found a moderate, statistically significant positive correlation between K and AST (r = 0.4529), and between Sb and ALT (r = 0.5241) in women with obesity. Furthermore, Pb showed a positive association with the liver enzyme GGT (r = 0.6263) in women with overweight. Our findings highlight the necessity of addressing obesity to reduce the risk of liver injury.

Li et al. [80] concluded that Pb, Cd, and Hg had inverse associations with the risk of peripheral or abdominal obesity. Similarly, a study by Rothenberg et al. [81] reported that blood Hg levels were also inversely related to BMI in adults. In our present study, serum Hg concentrations were 0.06 ± 0.02 ng/µL in women with overweight and 0.07 ± 0.03 ng/µL in women with obesity, using the CV-AAS method. Our findings did not reveal associations between Hg and biochemical parameters, or oxidative status markers linked with excess body weight. However, we noted a moderate positive correlation between Hg and Na (r = 0.5036; *p* < 0.05) in women with overweight. In the group of women with obesity, we detected a negative correlation analysis between Hg and K (r = −0.4605; *p* < 0.05). Women are more vulnerable to toxic elements compared to men due to differences in redox homeostasis processes, hormonal influences, and immunological responses between the sexes [82,83]. Furthermore, older individuals may have higher concentrations of these toxic elements compared to younger populations, a consequence of their accumulative effect on tissues and organs [84].

## 5. Conclusions

The determination of risk elements in blood matrices is an essential tool that provides information related to a variety of health outcomes. Our findings underscore the importance of addressing excess body weight and obesity in relation to essential, potentially toxic, and toxic elements. The present study revealed associations between serum concentrations of macro- and microelements and biochemistry markers in postmenopausal women with overweight/obesity. Notably, individuals who have obesity, as indicated by a higher BMI, percentage of body fat, and visceral fat area, had elevated serum levels of Ca, Mg, Fe, Al, Sr, Pb, and Hg. Sodium was the most frequently detected macroelement in both groups. Concentrations of Al, Cu, K, Sb, Zn, and Pb affected biochemical and liver function markers at multiple levels. Our results showed that Cu and Al were associated with increased serum total cholesterol. Furthermore, the correlation analysis between TAS and Cu, Al, and Ni confirmed associations in women with overweight/obesity. Our findings did not reveal associations between Hg and biochemical parameters or oxidative status markers. Additional prospective research could be valuable in understanding the consequences of element accumulation and biomarker changes, as well as in identifying potential targets for the treatment or prevention of comorbidities in people with obesity.

## Figures and Tables

**Table 1:** Parameters for the determination of elements using the ICP-OES technique.

Method Parameters/Units	
RF power (kW)	0.90
Plasma gas flow (L/min)	15.0
Auxiliary gas flow (L/min)	1.50
Nebulizer gas flow (L/min)	1.0
Replicated read time (s)	3.0
Instrument stabilization (s)	20.0
Sample uptake delay (s)	25.0
Pump rate (rpm)	15.0
Rinse time (s)	20.0
CCD detector temperature (°C)	−35
Element (λ/nm)	Ag 328.068; Al 167.019; As 188.980; Ba 455.403; Ca 315.887; Cd 226.502; Co 228.615; Cr 267.716; Cu 324.754; Fe 234.350; K 766.491; Li 670.783; Mg 383.829; Mn 257.610; Na 589.592; Ni 231.604; Pb 220.353; Sb 206.834; Se 196.026; Sr 407.771; Zn 206.200

**Table 2:** Baseline descriptive characteristics of the study group (*n* = 48).

Parameters/ Units	Standard/Optimal Reference Range for Adult	Women (*n* = 48)								
		Overweight (*n* = 26)				Obesity (*n* = 22)				*p*-Value
		x ± SD	min	max	CV (%)	x ± SD	min	max	CV (%)	
Age (years)	-	56.92 ± 4.20	50.00	64.00	7.39	56.91 ± 3.89	50.00	63.00	6.84	ns
**Anthropometric parameters**										
Body height (cm)	-	166.30 ± 5.82	156.00	180.00	3.50	163.20 ± 6.04	151.00	174.00	3.70	ns
Body weight (kg)	-	**75.86 ± 5.55**	63.50	86.80	7.31	**90.12 ± 10.86**	70.80	113.75	12.05	**<0.001**
BMI (≥25–29.90 kg/m^2^)	18.50–24.90 ^1^	**27.43 ± 1.37**	25.11	29.72	4.98					**<0.001**
BMI (≥30 kg/m^2^)						**33.76 ± 2.81**	30.27	39.47	8.31	**<0.001**
PBF (%)	18.0–28.0 ^2^	**38.74 ± 2.83**	33.88	43.84	7.31	**44.86 ± 4.31**	35.70	50.69	9.60	**<0.001**
VFA (cm^2^)	<100 ^2^	**120.03 ± 13.08**	97.91	144.03	10.88	**148.90 ± 18.97**	116.31	188.78	12.75	**<0.001**
**Blood pressure**										
Systolic blood pressure (mm Hg)	<120	130.00 ± 13.47	105.50	157.00	10.36	130.14 ± 12.38	106.00	150.50	9.52	ns
Diastolic blood pressure (mm Hg)	<80	85.85 ± 6.75	70.50	102.50	7.86	87.30 ± 6.63	75.00	101.00	7.59	ns

BMI—body mass index (kg/m^2^); PBF—percentage of body fat (%); VFA—visceral fat area (cm^2^); x—arithmetic mean; ±SD—standard deviation; min—minimum; max—maximum; CV (%)—coefficient of variation; Bold values are statistically significant; ns—not significant; ^1^ BMI classified according to the standards of the WHO [21,22]; ^2^ InBody 720—the precision body composition analyzer (user’s manual) (Biospace Co., Ltd., Seoul, Republic of Korea) [44], the optimal reference range is presented on the result sheet by the analyzer.

**Table 3:** The selected biochemical, enzymatic, and oxidative status parameters of blood serum in women with overweight/obesity.

Parameters/ Units	Standard/Optimal Reference Range for Adult	Women (*n* = 48)								
		Overweight (*n* = 26)				Obesity (*n* = 22)				*p*-Value
		x ± SD	min	max	CV (%)	x ± SD	min	max	CV (%)	
**Biochemical parameters**										
TC (mmol/L)	<5.20 ^1^	6.17 ± 1.04	4.37	8.49	16.85	6.58 ± 0.91	5.34	8.59	13.90	ns
Glucose (mmol/L)	3.90–6.10 ^2^	5.17 ± 0.56	4.09	6.35	10.81	5.35 ± 0.60	4.62	7.06	11.17	ns
tbil (µmol/L)	1.70–21.0 ^2^	9.16 ± 3.06	3.79	15.42	33.42	8.63 ± 1.87	4.65	12.38	21.67	ns
dbil (µmol/L)	<3.40 ^2^	3.07 ± 0.87	1.70	5.25	28.42	2.86 ± 0.55	1.73	4.04	19.16	ns
**Liver enzymatic parameters**										
AST (µkat/L)	<0.52 ^3^	0.33 ± 0.08	0.23	0.55	25.94	0.34 ± 0.10	0.25	0.74	30.29	ns
ALT (µkat/L)	<0.57 ^4^	**0.30 ± 0.09**	0.18	0.57	30.17	**0.39 ± 0.15**	0.20	0.76	37.98	**0.010**
GGT (µkat/L)	<0.63 ^5^	**0.40 ± 0.20**	0.21	0.93	49.98	**0.53 ± 0.22**	0.24	0.97	41.27	**0.036**
**Oxidative parameters**										
TAS (mmol/L)	1.30–1.77 ^6^	1.72 ± 0.13	1.42	1.98	7.70	1.71 ± 0.13	1.41	1.95	7.84	ns

TC—total cholesterol (mmol/L); tbil—total bilirubin (µmol/L); dbil—direct/conjugated bilirubin (µmol/L); AST—aspartate aminotransferase (µkat/L); ALT—alanine aminotransferase (µkat/L); GGT—γ-glutamyltransferase (µkat/L); TAS—total antioxidant status (mml/L); x—arithmetic mean; ±SD—standard deviation; min—minimum; max—maximum; CV (%)—coefficient of variation; Bold values are statistically significant; ns—not significant; ^1^ [45]; ^2^ [47]; ^3,4^ [48,49]; ^5^ [50]; ^6^ [46].

**Table 4:** The concentration of the macro- and microelements in the blood serum of women who were overweight/obesity.

Elements/λ (nm)	Women (*n* = 48)		
	Overweight (*n* = 26)	Obesity (*n* = 22)	*p*-Value
	x ± SD	x ± SD	
Al mg/mL/167.019	1.19 ± 0.92	1.23 ± 0.87	ns
Ba mg/mL/455.403	0.62 ± 0.15	0.61 ± 0.14	ns
Ca mg/mL/315.887	157.39 ± 18.89	162.51 ± 19.98	ns
Cu mg/mL/324.754	1.12 ± 0.11	1.09 ± 0.17	ns
Fe mg/mL/234.350	1.31 ± 0.36	1.36 ± 0.61	ns
K mg/mL/766.491	159.78 ± 10.81	159.33 ± 15.82	ns
Mg mg/mL/383.829	3.57 ± 0.45	3.73 ± 0.48	ns
Na mg/mL/589.592	2558.87 ± 102.37	2555.03 ± 175.78	ns
Ni mg/mL/231.604	0.14 ± 0.12	0.08 ± 0.06	ns
Pb mg/mL/220.353	0.14 ± 0.04	0.15 ± 0.06	ns
Sb mg/mL/206.834	0.22 ± 0.07	0.21 ± 0.07	ns
Se mg/mL/196.026	0.44 ± 0.15	0.40 ± 0.15	ns
Sr mg/mL/407.771	0.67 ± 0.18	0.72 ± 0.14	ns
Zn mg/mL/206.200	0.99 ± 0.33	0.93 ± 0.26	ns
Hg ng/µL ^1^/253.65	0.06 ± 0.02 ^1^	0.07 ± 0.03 ^1^	ns
Ag (328.068); As (188.980); Cd (226.502); Co (228.615); Cr (267.716); Li (670.783); Mn (257.610)	nd		

x—arithmetic mean; ±SD—standard deviation; nd—not detected; ns—not significant. ^1^ Concentration of Hg in blood serum was determined by CV-AAS on the AMA 254 Hg analyzer. Values were reported in units of ng/µL.

**Table 5:** Spearman correlation of biochemical and liver enzymatic parameters in women who are overweight.

	Glucose	TC	tbil	dbil	AST	ALT	GGT
TC	0.2628						
tbil	0.0186	0.4575 *					
dbil	−0.2638	0.2791	0.9139 ***				
AST	0.4529 *	−0.1155	−0.4434 *	−0.4781 *			
ALT	0.5334 **	−0.3127	−0.2491	−0.2715	0.5693 **		
GGT	−0.0175	−0.2582	−0.3652	−0.2390	0.1514	0.3436	
TAS	0.1418	−0.0777	0.2118	0.1274	−0.0488	0.3626	0.3981

TC—total cholesterol; tbil—total bilirubin; dbil—direct/conjugated bilirubin; AST—aspartate aminotransferase; ALT—alanine aminotransferase; GGT—γ-glutamyltransferase; TAS—total antioxidant status; *** (*p* < 0.001); ** (*p* < 0.01); * (*p* < 0.05).

**Table 6:** Spearman correlation of biochemical and liver enzymatic parameters in women with obesity.

	Glucose	TC	tbil	dbil	AST	ALT	GGT
TC	0.0403						
tbil	0.2400	0.0909					
dbil	0.2196	−0.0097	0.8916 ***				
AST	0.0097	−0.2390	0.2430	0.2749			
ALT	0.1515	−0.1385	0.0735	0.0754	0.7652 ***		
GGT	0.1468	0.0489	0.1306	0.1325	0.4348	0.4194	
TAS	0.3445	0.2145	0.2645	0.2216	−0.1250	−0.2691	−0.2296

TC—total cholesterol; tbil—total bilirubin; dbil—direct/conjugated bilirubin; AST—aspartate aminotransferase; ALT—alanine aminotransferase; GGT—γ-glutamyltransferase; TAS—total antioxidant status; *** (*p* < 0.001).

**Table 7:** Spearman correlation of macro- and microelements in the blood serum of women who are overweight.

	Ca	Na	K	Mg	Al	Ba	Cu	Fe	Ni	Pb	Sr	Zn	Se	Sb
Na	0.3246													
K	0.2592	0.1954												
Mg	0.9285 ***	0.4523 *	0.2254											
Al	0.7623 **	−0.0294	−0.1054	0.6103 **										
Ba	0.6254 **	0.0192	0.1862	0.5785 **	0.3578									
Cu	0.0989	−0.1808	0.3455	0.0246	0.0368	0.2566								
Fe	−0.0108	0.3015	−0.1754	0.0177	0.0931	−0.0885	−0.2524							
Ni	0.0343	0.2377	0.0074	−0.0221	−0.0545	−0.5833 *	−0.2170	0.5049 *						
Pb	0.0797	−0.1667	0.0034	0.0463	0.1716	−0.1322	−0.1492	0.1379	0.3964					
Sr	0.9515 ***	0.2615	0.2185	0.8777 ***	0.6789 **	0.6623 **	0.0854	−0.1446	−0.1054	0.0667				
Zn	−0.0054	−0.1946	−0.1762	0.0077	−0.0809	−0.0808	−0.0308	0.1800	0.4093	0.1904	−0.1292			
Se	−0.1814	−0.0618	0.1388	−0.0753	−0.6152 **	−0.0122	−0.3430	−0.0035	−0.1589	−0.0023	−0.0874	−0.0383		
Sb	0.4647 *	0.2407	0.1058	0.4973 *	0.3299	0.3183	0.2932	−0.1404	−0.1626	−0.2323	0.4607 *	0.1720	−0.3792	
Hg	0.1774	0.5036 *	0.0004	0.2427	0.1029	0.2553	0.0217	0.3505	−0.0618	0.0725	0.1796	−0.2909	−0.3136	0.4114

*** (*p* < 0.001); ** (*p* < 0.01); * (*p* < 0.05).

**Table 8:** Spearman correlation of macro- and microelements in the blood serum of women with obesity.

	Ca	Na	K	Mg	Al	Ba	Cu	Fe	Ni	Pb	Sr	Zn	Se	Sb
Na	0.7764 ***													
K	0.3665	0.4828 *												
Mg	0.8938 ***	0.7154 ***	0.3371											
Al	0.2714	0.2652	−0.1166	0.2487										
Ba	0.5381 **	0.2445	0.1327	0.6014 **	−0.2921									
Cu	0.3988	0.2056	−0.0006	0.4213	0.2570	−0.0260								
Fe	0.2106	0.0446	0.4907 *	0.1711	−0.4510	0.0785	−0.0768							
Ni	0.1226	−0.0245	0.2277	0.1051	0.4316	−0.2802	0.6410 *	0.4413						
Pb	0.0209	−0.2129	−0.1112	−0.0740	0.0444	−0.0096	0.2480	0.0920	0.4448					
Sr	0.9187 ***	0.7290 ***	0.3179	0.9176 ***	0.2198	0.6657 **	0.2649	0.0480	−0.0105	−0.0503				
Zn	0.5508 **	0.3836	0.3780	0.5085 *	0.1880	0.3130	0.0463	0.4119	0.0561	0.1407	0.4192			
Se	−0.6219 **	−0.4637 *	−0.3581	−0.7365 ***	0.0650	−0.6224 **	−0.1833	−0.1909	−0.1086	0.1666	−0.7060 ***	−0.2577		
Sb	0.3187	0.3696	0.1580	0.4996 *	0.0822	0.1958	0.1419	0.3266	0.6276	0.0667	0.3766	0.2512	−0.4756 *	
Hg	−0.2696	−0.1897	−0.4605 *	−0.1864	0.1913	−0.1546	0.0474	−0.3020	0.0729	−0.0195	−0.1559	−0.2576	0.1020	0.3096

*** (*p* < 0.001); ** (*p* < 0.01); * (*p* < 0.05).

**Table 9:** Statistically significant correlations between risk elements and all investigated biomarkers in the blood serum of women with overweight/obesity.

Women (*n* = 48)					
Overweight (*n* = 26)			Obesity (*n* = 22)		
Investigated Parameter	Element	Spearman *R* (*p*-Value)	Investigated Parameter	Element	Spearman *R* (*p*-Value)
Glucose	Sb	−0.4451 (0.0465)	TC	Cu	0.5530 (0.0134)
TC	Al	0.5441 (0.0351)	AST	K	0.4529 (0.0428)
tbil	Zn	−0.5400 (0.0096)	ALT	Sb	0.5241 (0.0307)
dbil	Zn	−0.5243 (0.0119)	TAS	Al	0.5939 (0.0175)
GGT	Pb	0.6263 (0.0079)	TAS	Ni	0.6485 (0.0315)
TAS	Cu	0.4055 (0.0470)	-	-	-

TC—total cholesterol; tbil—total bilirubin; dbil—direct/conjugated bilirubin; AST—aspartate aminotransferase; ALT—alanine aminotransferase; GGT—γ-glutamyltransferase; TAS—total antioxidant status.

## Data Availability

All datasets related to the results and consequences of the study are available from the corresponding author (M.H.) upon reasonable request.

## References

[1] Soylak M., Aydin A. (2011). Determination of some heavy metals in food and environmental samples by flame atomic absorption spectrometry after coprecipitation. Food Chem. Toxicol..

[2] Briffa J., Sinagra E., Blundell R. (2020). Heavy metal pollution in the environment and their toxicological effects on humans. Heliyon.

[3] Balali-Mood M., Naseri K., Tahergorabi Z., Khazdair M.R., Sadeghi M. (2020). Toxic Mechanisms of Five Heavy Metals: Mercury, Lead, Chromium, Cadmium, and Arsenic. Front. Pharmacol..

[4] Kňažická Z., Ďúranová H., Fialková V., Bilčíková J., Lukáč N. (2021). Potentially Toxic Elements in Relation to Sexual Steroid Hormones.

[5] Massanyi P., Stawarz R., Halo M., Formicki G., Lukac N., Cupka P., Schwarcz P., Kovacik A., Tusimova E., Kovacik J. (2014). Blood concentration of copper, cadmium, zinc and lead in horses and its relation to hematological and biochemical parameters. J. Environ. Sci. Health A Tox. Hazard Subst. Environ. Eng..

[6] Kovacik A., Tvrda E., Miskeje M., Arvay J., Tomka M., Zbynovska K., Andreji J., Hleba L., Kovacikova E., Fik M. (2019). Trace Metals in the Freshwater Fish *Cyprinus carpio*: Effect to Serum Biochemistry and Oxidative Status Markers. Biol. Trace Elem. Res..

[7] da Silva J.F., Williams R.J.P. (2001). The Biological Chemistry of the Elements.

[8] Soetan K.O., Olaiya C.O., Oyewole O.E. (2010). The Importance of Mineral Elements for Humans, Domestic Animals and Plants: A Review. Afr. J. Food Sci..

[9] Kamunda C., Mathuthu M., Madhuku M. (2016). Health Risk Assessment of Heavy Metals in Soils from Witwatersrand Gold Mining Basin, South Africa. Int. J. Environ. Res. Public Health.

[10] Celep G.S., Kaynar P., Rastmanesh R. (2017). Biochemical functions of micronutrients. Adv. Obes. Weight Manag. Control.

[11] Knazicka Z., Lukac N., Forgacs Z., Tvrdá E., Lukacova J., Slivkova J., Binkowski Ł., Massanyi P. (2013). Effects of mercury on the steroidogenesis of human adrenocarcinoma (NCI-H295R) cell line. J. Environ. Sci. Health A Tox. Hazard Subst. Environ. Eng..

[12] Knazicka Z., Forgacs Z., Lukacova J., Roychoudhury S., Massanyi P., Lukac N. (2015). Endocrine disruptive effects of cadmium on steroidogenesis: Human adrenocortical carcinoma cell line NCI-H295R as a cellular model for reproductive toxicity testing. J. Environ. Sci. Health A Tox. Hazard Subst. Environ. Eng..

[13] Keil D.E., Berger-Ritchie J., McMillin G.A. (2011). Testing for toxic elements: A focus on arsenic, cadmium, lead and mercury. Labmedicine.

[14] Harrington J.M., Young D.J., Essader A.S., Sumner S.J., Levine K.E. (2014). Analysis of human serum and whole blood for mineral content by ICP-MS and ICP-OES: Development of a mineralomics method. Biol. Trace Elem. Res..

[15] Sun J., Ruan Y., Xu N., Wu P., Lin N., Yuan K., An S., Kang P., Li S., Huang Q. (2023). The effect of dietary carbohydrate and calorie restriction on weight and metabolic health in overweight/obese individuals: A multi-center randomized controlled trial. BMC Med..

[16] Frühbeck G., Toplak H., Woodward E., Yumuk V., Maislos M., Oppert J.M. (2013). Obesity: The gateway to ill health—An EASO position statement on a rising public health, clinical and scientific challenge in Europe. Obes. Facts.

[17] Németh Z., Siptár M., Tóth N., Tóth K., Csontos C., Kovács-Ábrahám Z., Csongor A., Molnár F., Márton Z., Márton S. (2023). Indications for Sleeve Gastrectomy—Is It Worth Waiting for Comorbidities to Develop?. Medicina.

[18] Chooi Y.C., Ding C., Magkos F. (2019). The epidemiology of obesity. Metabolism.

[19] Lobstein T., Brinsden H., Neveux M. World Obesity Atlas 2022. https://s3-eu-west-1.amazonaws.com/woffiles/World_Obesity_Atlas_2022.pdf.

[20] Obesity Prevalence 2019. https://data.worldobesity.org/country/slovakia-193/#data_prevalence.

[21] World Health Organization Obesity and Overweight. https://www.who.int/news-room/fact-sheets/detail/obesity-and-overweight.

[22] World Health Organisation A Healthy Lifestyle—WHO Recommendations. https://www.who.int/europe/news-room/fact-sheets/item/a-healthy-lifestyle---who-recommendations.

[23] Arnold M., Leitzmann M., Freisling H., Bray F., Romieu I., Renehan A., Soerjomataram I. (2016). Obesity and cancer: An update of the global impact. Cancer Epidemiol..

[24] Bihari M., Habánová M., Jančichová K., Gažarová M. (2022). Diagnosis of obesity and evaluation of the risk of premature death (ABSI) based on body mass index and visceral fat area. Rocz. Panstw. Zakl. Hig..

[25] Ranasinghe C., Gamage P.T., Katulanda P., Andraweera N.D., Thilakarathne S., Tharanga P. (2013). Relationship between Body Mass Index (BMI) and body fat percentage, estimated by bioelectrical impedance, in a group of Sri Lankan adults: A cross sectional study. BMC Public Health.

[26] Freedman D.S., Ogden C.L., Kit B.K. (2015). Interrelationships between BMI, skinfold thicknesses, percent body fat, and cardiovascular disease risk factors among U.S. children and adolescents. BMC Pediatr..

[27] Gažarová M., Galšneiderová M., Mečiarová L. (2019). Obesity diagnosis and mortality risk based on a body shape index (ABSI) and other indices and anthropometric parameters in university students. Rocz. Panstw. Zakl. Hig..

[28] Wu L., Zhu W., Qiao Q., Huang L., Li Y., Chen L. (2021). Novel and traditional anthropometric indices for identifying metabolic syndrome in non-overweight/obese adults. Nutr. Metab..

[29] Gažarová M., Bihari M., Lorková M., Lenártová P., Habánová M. (2022). The Use of Different Anthropometric Indices to Assess the Body Composition of Young Women in Relation to the Incidence of Obesity, Sarcopenia and the Premature Mortality Risk. Int. J. Environ. Res. Public Health.

[30] Zeng Q., Dong S.Y., Sun X.N., Xie J., Cui Y. (2012). Percent body fat is a better predictor of cardiovascular risk factors than body mass index. Braz. J. Med. Biol. Res..

[31] Trang L.T., Trung N.N., Chu D.T., Hanh N.T.H. (2019). Percentage Body Fat is As a Good Indicator for Determining Adolescents Who Are Overweight or Obese: A Cross-Sectional Study in Vietnam. Osong Public Health Res. Perspect..

[32] Liu H., Yang D., Li S., Xiao Y., Tu Y., Peng D., Bao Y., Han J., Yu H. (2022). A Reliable Estimate of Visceral Fat Area From Simple Anthropometric Measurements in Chinese Overweight and Obese Individuals. Front. Endocrinol..

[33] Blüher M. (2019). Obesity: Global epidemiology and pathogenesis. Nat. Rev. Endocrinol..

[34] Yumuk V., Tsigos C., Fried M., Schindler K., Busetto L., Micic D., Toplak H. (2015). Obesity Managemenet Task Force of the European Association for the Study of Obesity. European Guidelines for Obesity Management in Adults. Obes. Facts.

[35] McKay J., Ho S., Jane M., Pal S. (2020). Overweight & obese Australian adults and micronutrient deficiency. BMC Nutr..

[36] Khaodhiar L., McCowen K.C., Blackburn G.L. (1999). Obesity and its comorbid conditions. Clin. Cornerstone.

[37] Fruh S.M. (2017). Obesity: Risk factors, complications, and strategies for sustainable long-term weight management (Review). J. Am. Assoc. Nurse Pract..

[38] World Health Organization (2000). Obesity: Preventing and Managing the Global Epidemic: Report of a WHO Consultation.

[39] Lim Y., Boster J. (2023). Obesity and Comorbid Conditions.

[40] Habanova M., Holovicova M., Scepankova H., Lorkova M., Gazo J., Gazarova M., Pinto C.A., Saraiva J.A., Estevinho L.M. (2022). Modulation of Lipid Profile and Lipoprotein Subfractions in Overweight/Obese Women at Risk of Cardiovascular Diseases through the Consumption of Apple/Berry Juice. Antioxidants.

[41] Árvay J., Šnirc M., Hauptvogl M., Bilčíková J., Bobková A., Demková L., Hudáček M., Hrstková M., Lošák T., Král M. (2019). Concentration of Micro- and Macro-elements in green and roasted coffee: Influence of roasting degree and risk assessment for the consumers. Biol. Trace Elem. Res..

[42] Harangozo L., Šnirc M., Árvay J., Jakabová S., Čéryová S. (2021). Biogenic and risk elements in Walnuts (*Juglans regia* L.) from chosen localities of Slovakia. Biol. Trace Elem. Res..

[43] Faul F., Erdfelder E., Lang A.G., Buchner A. (2007). G*Power 3: A flexible statistical power analysis program for the social, behavioral, and biomedical sciences. Behav Res. Methods.

[44] Biospace: InBody 720—The Precision Body Composition Analyzer (User’s Manual). Seoul, South Korea. https://www.bodyanalyse.no/gammel/images/stories/inbody/dokumenter/InBody720_User_manual.pdf.

[45] Schaefer E.J., McNamara J., Rifai N., Warnick G.R., Dominiczak M.H. (1997). Overview of the diagnosis and treatment of lipid disorders. Handbook of Lipoprotein Testing.

[46] Miller N.J., Rice-Evans C., Davies M.J., Gopinathan V., Milner A. (1993). A novel method for measuring antioxidant capacity and its application to monitoring the antioxidant status in premature neonates. Clin. Sci..

[47] Thomas L. (1998). Clinical Laboratory Diagnostics.

[48] Schumann G., Bonora R., Ceriotti F., Férard G., Ferrero C.A., Franck P.F., Gella F.J., Hoelzel W., Jørgensen P.J., Kanno T. (2002). IFCC primary reference procedures for the measurement of catalytic activity concentrations of enzymes at 37 °C. International Federation of Clinical Chemistry and Laboratory Medicine. Part 5: Reference procedure for the measurement of catalytic concentration of aspartate aminotransferase. Clin. Chem. Lab. Med..

[49] Schumann G., Bonora R., Ceriotti F., Férard G., Ferrero C.A., Franck P.F., Gella F.J., Hoelzel W., Jørgensen P.J., Kanno T. (2002). IFCC primary reference procedures for the measurement of catalytic activity concentrations of enzymes at 37 °C. International Federation of Clinical Chemistry and Laboratory Medicine. Part 4: Reference procedure for the measurement of catalytic concentration of alanine aminotransferase. Clin. Chem. Lab. Med..

[50] Siekmann L., Bonora R., Burtis C.A., Ceriotti F., Clerc-Renaud P., Férard G., Ferrero C.A., Forest J.C., Franck P.F., Gella F.J. (2002). IFCC primary reference procedures for the measurement of catalytic activity concentrations of enzymes at 37 °C. International Federation of Clinical Chemistry and Laboratory Medicine. Part 7: Certification of four reference materials for the determination of enzymatic activity of gamma-glutamyltransferase, lactate dehydrogenase, alanine aminotransferase and creatine kinase accord. Clin. Chem. Lab. Med..

[51] Górnicka M., Szewczyk K., Białkowska A., Jancichova K., Habanova M., Górnicki K., Hamulka J. (2022). Anthropometric Indices as Predictive Screening Tools for Obesity in Adults; The Need to Define Sex-Specific Cut-Off Points for Anthropometric Indices. Appl. Sci..

[52] Jeon H.H., Lee Y.K., Kim D.H., Pak H., Shin S.Y., Seo J.H. (2021). Risk for metabolic syndrome in the population with visceral fat area measured by bioelectrical impedance analysis. Korean J. Intern. Med..

[53] Zając-Gawlak I., Kłapcińska B., Kroemeke A., Pośpiech D., Pelclová J., Přidalová M. (2017). Associations of visceral fat area and physical activity levels with the risk of metabolic syndrome in postmenopausal women. Biogerontology.

[54] World Health Organization Mortality and Burden of Disease Attributable to Selected Major Risks. https://www.who.int/publications/i/item/9789241563871.

[55] Banach W., Nitschke K., Krajewska N., Mongiałło W., Matuszak O., Muszyński J., Skrypnik D. (2020). The Association between Excess Body Mass and Disturbances in Somatic Mineral Levels. Int. J. Mol. Sci..

[56] Zhang X., Wang J., Li J., Yu Y., Song Y.A. (2018). A positive association between dietary sodium intake and obesity and central obesity: Results from the National Health and Nutrition Examination Survey 1999–2006. Nutr. Res..

[57] Lanaspa M.A., Kuwabara M., Andres-Hernando A., Li N., Cicerchi C., Jensen T., Orlicky D.J., Roncal-Jimenez C.A., Ishimoto T., Nakagawa T. (2018). High salt intake causes leptin resistance and obesity in mice by stimulating endogenous fructose production and metabolism. Proc. Natl. Acad. Sci. USA.

[58] Morais J.B., Severo J.S., Santos L.R., de Sousa Melo S.R., de Oliveira Santos R., de Oliveira A.R., Cruz K.J., do Nascimento Marreiro D. (2017). Role of Magnesium in Oxidative Stress in Individuals with Obesity. Biol. Trace Elem. Res..

[59] Chen J.M., Wu T.Y., Wu Y.F., Kuo K.L. (2023). Association of the serum calcium level with metabolic syndrome and its components among adults in Taiwan. Arch. Endocrinol. Metab..

[60] Ren X.H., Yao Y.S., He L.P., Jin Y.L., Chang W.W., Li J., Chen Y., Song X.L., Tang H., Ding L.L. (2013). Overweight and obesity associated with increased total serum calcium level: Comparison of cross-sectional data in the health screening for teaching faculty. Biol. Trace Elem. Res..

[61] Zohal M., Jam-Ashkezari S., Namiranian N., Moosavi A., Ghadiri-Anari A. (2019). Association between selected trace elements and body mass index and waist circumference: A cross sectional study. Diabetes Metab. Syndr..

[62] Mainous A.G., Wright R.U., Hulihan M.M., Twal W.O., McLaren C.E., Diaz V.A., McLaren G.D., Argraves W.S., Grant A.M. (2014). Elevated transferrin saturation, health-related quality of life and telomere length. Biometals.

[63] Tokuda E., Okawa E., Watanabe S., Ono S. (2014). Overexpression of metallothionein-I, a copper-regulating protein, attenuates intracellular copper dyshomeostasis and extends lifespan in a mouse model of amyotrophic lateral sclerosis caused by mutant superoxide dismutase-1. Hum. Mol. Genet..

[64] Lecube A., Carrera A., Losada E., Hernández C., Simó R., Mesa J. (2006). Iron deficiency in obese postmenopausal women. Obesity.

[65] Menzie C.M., Yanoff L.B., Denkinger B.I., McHugh T., Sebring N.G., Calis K.A., Yanovski J.A. (2008). Obesity-related hypoferremia is not explained by differences in reported intake of heme and nonheme iron or intake of dietary factors that can affect iron absorption. J. Am. Diet. Assoc..

[66] Alshwaiyat N.M., Ahmad A., Wan Hassan W.M.R., Al-Jamal H.A.N. (2021). Association between obesity and iron deficiency (Review). Exp. Ther. Med..

[67] Yang H., Liu C.N., Wolf R.M., Ralle M., Dev S., Pierson H., Askin F., Steele K.E., Magnuson T.H., Schweitzer M.A. (2019). Obesity is associated with copper elevation in serum and tissues. Metallomics.

[68] Habib S.A., Saad E.A., Elsharkawy A.A., Attia Z.R. (2015). Pro-inflammatory adipocytokines, oxidative stress, insulin, Zn and Cu: Interrelations with obesity in Egyptian non-diabetic obese children and adolescents. Adv. Med. Sci..

[69] Mailloux R.J., Lemire J., Appanna V.D. (2011). Hepatic response to aluminum toxicity: Dyslipidemia and liver diseases (Review). Exp. Cell Res..

[70] Bignucolo A., Lemire J., Auger C., Castonguay Z., Appanna V., Appanna V.D. (2012). The Molecular Connection between Aluminum Toxicity, Anemia, Inflammation and Obesity: Therapeutic Cues.

[71] Parris W.E., Adeli K., Sarkar B. (2002). In Vitro toxicological assessment of heavy metals and intracellular mechanisms of toxicity. Heavy Metals in the Environment.

[72] Shi X., Dalal N.S., Kasprzak K.S. (1993). Generation of free radicals in reactions of Ni (II)-thiol complexes with molecular oxygen and model lipid hydroperoxides. J. Inorg. Biochem..

[73] Valko M., Morris H., Cronin M.T.D. (2005). Metals, toxicity and oxidative stress. Curr. Med. Chem..

[74] Fu Z., Xi S. (2020). The effects of heavy metals on human metabolism. Toxicol. Mech. Methods.

[75] Kipp Z.A., Xu M., Bates E.A., Lee W.H., Kern P.A., Hinds T.D. (2023). Bilirubin Levels Are Negatively Correlated with Adiposity in Obese Men and Women, and Its Catabolized Product, Urobilin, Is Positively Associated with Insulin Resistance. Antioxidants.

[76] Mota Martins L., Soares de Oliveira A.R., Clímaco Cruz K.J., Borges de Araújo C.G., de Oliveira F.E., Santos de Sousa G., do Nascimento Nogueira N., do Nascimento Marreiro D. (2014). Influence of corticol on zinc metabolism in morbidly obese women. Nutr. Hosp..

[77] Payahoo L., Ostadrahimi A., Mobasseri M., Khaje Bishak Y., Farrin N., Asghari Jafarabadi M., Mahluji S. (2013). Effects of zinc supplementation on the anthropometric measurements, lipid profiles and fasting blood glucose in the healthy obese adults. Adv. Pharm. Bull..

[78] Kim D.W., Ock J., Moon K.W., Park C.H. (2021). Association between Pb, Cd, and Hg Exposure and Liver Injury among Korean Adults. Int. J. Environ. Res. Public Health.

[79] Li W., Li X., Su J., Chen H., Zhao P., Qian H., Gao X., Ye Q., Zhang G., Li X. (2023). Associations of blood metals with liver function: Analysis of NHANES from 2011 to 2018. Chemosphere.

[80] Li T., Yu L., Yang Z., Shen P., Lin H., Shui L., Tang M., Jin M., Chen K., Wang J. (2022). Associations of Diet Quality and Heavy Metals with Obesity in Adults: A Cross-Sectional Study from National Health and Nutrition Examination Survey (NHANES). Nutrients.

[81] Rothenberg S.E., Korrick S.A., Fayad R. (2015). The influence of obesity on blood mercury levels for U.S. non-pregnant adults and children: NHANES 2007–2010. Environ. Res..

[82] Rechtman E., Curtin P., Papazaharias D.M., Renzetti S., Cagna G., Peli M., Levin-Schwartz Y., Placidi D., Smith D.R., Lucchini R.G. (2020). Sex-specific associations between co-exposure to multiple metals and visuospatial learning in early adolescence. Transl. Psychiatry.

[83] Gade M., Comfort N., Re D.B. (2021). Sex-specific neurotoxic effects of heavy metal pollutants: Epidemiological, experimental evidence and candidate mechanisms. Environ. Res..

[84] Chang L., Shen S., Zhang Z., Song X., Jiang Q. (2018). Study on the relationship between age and the concentrations of heavy metal elements in human bone. Ann. Transl. Med..

